# Men’s Facial Hair Preferences Reflect Facial Hair Impression Management Functions Across Contexts and Men Know It

**DOI:** 10.1007/s10508-023-02595-6

**Published:** 2023-04-17

**Authors:** Łukasz Jach, Marcin Moroń, Peter K. Jonason

**Affiliations:** 1 Institute of Psychology, Faculty of Social Sciences, University of Silesia in Katowice, Grażyńskiego Street 53, 40-126 Katowice, Poland; 2 Department of General Psychology, University of Padua, Padua, Italy; 3 Cross-Cultural Psychology Centre, Cardinal Stefan Wyszyński University, Warsaw, Poland

**Keywords:** Facial hair, Impression management, Intrasexual competition, Social perception, Social situations

## Abstract

Men with distinct facial hair are perceived as more aggressive, dominant, older, and more formidable, mostly by other men. However, despite considerable interest in the social perception of beardedness, only a few studies have explored men’s preferences toward facial hair. We investigated men’s preferences toward facial hair and whether their judgments of facial hair appropriateness in social situations depended on their actual beardedness and the type of social interaction (*N* = 509; age: *M* = 29.35, *SD* = 7.24). Men preferred having more facial hair, particularly when they have a heavy stubble or a full beard. Men preferred more facial hair for themselves compared to other men, suggesting that men’s preferences for facial hair could be based on the frequency-dependent value of a display, namely more value assigned to rarer attributes. Men’s judgments of the appropriateness of facial hair were sensitive to social situations for displaying facial hair as well. More facial hair was considered more appropriate for informal situations, especially with another man. Social norms associated with formal interaction could, therefore, limit the impression management functions of beards in intrasexual competition.

**Supplementary Information:**

The online version contains supplementary material available at 10.1007/s10508-023-02595-6.

## Introduction

Men and women differ markedly in the amount of visible facial hair they have (Lassek & Gaulin, 2022). While facial hair is virtually absent among women, it appears in men during puberty and is driven by the activation of androgens (Randall, 1994, 2008). Such dimorphic traits can perform both intersexual (e.g., enhancing sexual attractiveness) and intrasexual functions (e.g., intimidating rivals and enhancing social status; Dixson et al., 2005; Grueter et al., 2015). Thus, we consider facial hair as a display produced by an individual that functions to influence the behavior of other people. There is phylogenetic evidence that facial hair among men is a sexually selected secondary sexual trait. Men rank similarly in visually conspicuous secondary trait development (e.g., beards and patterned baldness) to male nonhuman primates with polygynous mating systems (Dixson et al., 2005) and large social group sizes with multilevel social organizations (Grueter et al., 2015). As a secondary sexual trait, men’s beards could affect the social perception of their age, social status, and dominance, all of which are primarily important in male-male competition and secondarily in attractiveness to females.

As facial hair is related to the level of androgens (Randall, 1994, 2008) it may enable adult men to display their sexual maturity to enhance their intersexual attractiveness. Facial hair augments observers’ perceptions of men’s maturity and masculinity (Addison, 1989; Neave & Shields, 2008). However, evidence that facial hair determines men’s sexual attractiveness is largely equivocal, where some studies reported facial hair to enhance men’s attractiveness (Dixson et al., 2018a, 2018b; McIntosh et al., 2017) while others do not (Dixson & Vasey, 2012; Dixson et al., 2013; Jach & Moroń, 2020; Muscarella & Cunningham, 1996). For instance, beardedness enhances men’s attractiveness rated by women when judging long-term over short-term relationships (Clarkson et al., 2020; Stower et al., 2020) and when considering fathering abilities than sexual attractiveness (Dixson & Brooks, 2013), particularly among mothers with young children (Dixson et al., 2019a, b).

### The Role of Facial Hair in Intrasexual Competition Among Men

Although the intersexual functionality of facial hair is ambiguous, facial hair seems to have clearer functions in intrasexual competition. Men compete with other men for partners and resources to acquire and keep partners (Arnocky & Carré, 2016), such as wealth and social status (Buss, 2006), and tend to be more aggressive toward a same-sex rival when their mate value is threatened (Bird et al., 2016). Men’s intrasexual rivalry ranges from direct combat (i.e., trying to physically dominate a rival), through verbal derogation of competitors, to noncombative self-promotion (i.e., trying to enhance positive qualities; Arnocky & Carré, 2016; Buss & Dedden, 1990).

The function of facial hair in men could differ as a function of the level of intrasexual competition in a situation. Soft, hairy surfaces can absorb impacts better than sheared surfaces, so facial hair is considered to reduce the risk of severe jaw damage in direct physical fights among men (Beseris et al., 2020). However, the costs of a direct physical confrontation could be detrimental for men involved in intrasexual rivalry (Wilson & Daly, 1985). Thus, an array of deterring displays may have evolved to gain an advantage in intrasexual competition while minimizing the costs associated with outright, physical competition (Arnocky & Carré, 2016). Manipulation of these displays could change the evaluation of such characteristics of their owners as combat skills, social status, alliances, and the possibility to inflict costs on a rival (Craig et al., 2019; Sell et al., 2014). Deterring displays directed to other men could function to gain a better social position and dominate other men (Albert et al., 2020) but also as a form of mate-guarding behavior that is meant to scare away potential rivals from mating with one’s sexual partner (Buss, 2002).

Beardedness could function as a deterring display that affects men’s dominance evaluation in intrasexual competition (Albert et al., 2020). When people look at bearded faces, they detect angry facial expressions faster and more accurately than when they look at clean-shaven faces (Craig et al., 2019; Dixson et al., 2021). Additionally, facial hair augments explicit aggressiveness ratings of angry facial expressions (Dixson & Vasey, 2012). Moreover, beards enhance judgments of men’s facial masculinity, dominance, and aggressiveness compared to clean-shaven faces (Dixson et al., 2017; Mefodeva et al., 2020; Sherlock et al., 2017). Men with thicker beards are perceived as physically stronger (Fink et al., 2006). However, some results suggest that facial hair may not be an accurate predictor of characteristics possessed by its owner. People perceive feminized faces with facial hair as more masculine than masculinized clean-shaven faces (Mefodeva et al., 2020). This suggests that facial hair may have a compensatory function when other elements of the masculine face appearance are less prominent (Mogilski & Welling, 2018). Facial hair does not predict either fighting success during direct agonistic contests among men (Dixson et al., 2018a, b; Třebický et al., 2019) or fighters’ physical strength (Třebický et al., 2019); however, people perceive men with more facial hair as better fighters than men with less facial hair (Třebický et al., 2019).

### Contextual Influences on Men’s Facial Hair Displaying Behaviors

Intrasexual competitive tactics may vary depending on several contextual factors such as the number of mates available locally or men’s relative mate value (Arnocky & Carré, 2016). The strong role of facial hair on perceptions of male dominance, aggressiveness, and masculinity suggests that men may opt to be more bearded under conditions of high intra-sexual competition. Beards are more frequent among men living in countries with male-biased sex ratios, lower health, higher pathogens, and greater economic disparity (Dixson & Lee, 2020; Dixson et al., 2019a, 2019b; Pazhoohi & Kingstone, 2020). On the other hand, deterring intrasexual functions of facial hair may be especially important in today's crowded urban environment, where a high frequency of contact with strangers requires quick self-presentation and quick recognition of other people's intentions (Dixson et al., 2017). Moreover, men could be aware of the beneficial role of facial hair in noncombative intrasexual rivalry thus they displayed a clear preference to have more facial hair for themselves compared to other men (Jach & Moroń, 2020).

Beardedness may play different functions for female observers (Neave & Shields, 2008) compared to male observers (Sherlock et al., 2017). Moreover, social interactions could have formal (e.g., job interview, business interaction) or informal characteristics (e.g., conversation in a pub; Giacalone & Rosenfeld, 1986). The social norms associated with a particular type of situation may create an additional context for intrasexual self-promotion. Regarding formal situations, for example, during hiring decisions, employers may associate wearing a beard with higher competence and more favorable personality traits (Reed & Blunk, 1990) but also lower conformity to rules (de Souza et al., 2003). In hospitality jobs (e.g., hotel attendants, restaurant service providers) beardedness leads to lower guests’ assurance attributions of an employee (Kim et al., 2017; Magnini et al., 2013). Beardedness of an actor is beneficial in advertising products that are associated with expertise and trustworthiness (Guido et al., 2011), but in political marketing could be linked with higher conservatism (Herrick et al., 2015). On the other hand, studies in which the assessment of men wearing beards compared to clean-shaven was conducted in informal circumstances demonstrated that wearing facial hair was perceived as associated with being enthusiastic, sincere, generous, extroverted, masculine, inquisitive, and stronger (Kenny & Fletcher, 1973; Pellegrini, 1973). These findings indicate that the impression management functions of facial hair could be more pronounced in informal (free of specific display rules) encounters with other people.

### The Present Study

Given the vast configurations of beards men can potentially grow (e.g., goatee, light stubble), men can manipulate their facial hair to accentuate or mitigate their intersexual attractiveness or intrasexual status. In the present study, we wanted to examine men’s preferences for facial hair controlling the level of participants’ actual facial hair and using realistic images of different levels of facial hair. Considering the intrasexual competition aspects, we predict that men would prefer more facial hair for themselves than for other men (H1) and that their actual facial hair would not be related to the amount of facial hair that is considered attractive to women (H2).

However, beards are subject to socio-cultural display rules (Oldstone-Moore, 2015), and enhancing or mitigating intrasexual competitive displays may depend on the type of social situation (de Sauza et al., 2003). Therefore, we also wanted to examine men’s judgments of facial hair appropriateness in interactions with unknown men and women in formal as opposed to informal contexts to assess how social norms influence competitive display. We predict that men would consider facial hair more appropriate in interactions with unknown men than with unknown women (H3). However, given that men with more facial hair are perceived as more aggressive and dominant (Neave & Shields, 2008), we predict that men would consider facial hair more appropriate in informal interactions (when there are no established rules of behavior) than in formal interactions (when there are established rules of behavior; H4). In addition, to ensure that our results are robust across at least two sample groups—convenience and paid participants—we treat sampling method as an additional factor in our analyses, although we have no predictions about differences therein.

## Method

### Participants and Procedure

A sample of 509 Polish men aged 18–57 (*M* = 29.35, *SD* = 7.24) consented to participate in an anonymous, online study via Lime Survey and SW Research survey platforms. Men recruited via Lime Survey (*n* = 95) were a convenience sample who participated without remuneration and men recruited via SW Research consisted of a more rigorous, heterosexual, internet panel sample who participated in exchange for points that could be exchanged for prizes. The participants were informed of the topic of the study and its length, and if they consented, they provided information about (1) their actual facial hair, (2) their most wanted facial hair, (3) facial hair most preferred for other men, and (4) facial hair most attractive for women. Participants also reported which facial hair was most appropriate to wear in (5) a formal situation with an unknown man, (6) a formal situation with an unknown woman, (7) an informal situation with an unknown man, and (8) an informal situation with an unknown woman. Sensitivity analysis indicated that the sample size was large enough to detect a small effect size (*f* = 0.16 which is an equivalent of η_p_^2^ = 0.025) with appropriate power (1-β = 0.80) given α equal to 0.05 (Faul et al., 2007).

### Measures

**Fig. 1:**
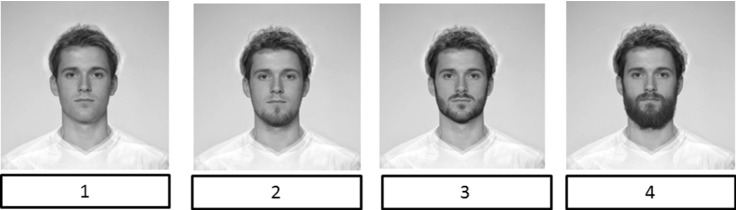
Morphed faces showing different types of facial hair. *Note*: 1 = “Clean-shaven”, 2 = “Light stubble”, 3 = “Heavy stubble”, 4 = “Full beard”

We used four specially designed photos of faces with different amounts of facial hair to create a pictorial scale of continuous preference for facial hair (see Fig. 1). We prepared the stimuli photos using the FaceApp application used to add various amounts of facial hair to the morphed average face of a white young adult male provided by DeBruine (2016). Men assessed their preferences for facial hair on the pictorial scale ranging from *cleanly shaven* (1) to *fully bearded* (4). Table 1 provides information on the frequency of each facial hair preference level indicated by the participants in the study.

**Table 1:** Counts and frequencies of facial hair indicated by participants

Facial hair	Clean shaven	Light stubble	Heavy stubble	Full beard
Actual	228 (44.79%)	102 (20.04%)	132 (25.93%)	47 (9.23%)
Most wanted for oneself	173 (33.99%)	77 (15.13%)	169 (33.20%)	90 (17.68%)
Preferred for other men	179 (35.17%)	109 (21.42%)	178 (34.97%)	43 (8.45%)
Perceived as attractive for women	133 (26.13%)	68 (13.36%)	250 (49.12%)	58 (11.39%)
*Most appropriate for…*				
… formal situation with an unknown man	271 (53.24%)	71 (13.95%)	137 (26.92%)	30 (5.89%)
… formal situation with an unknown woman	217 (42.63%)	88 (17.29%)	167 (32.81%)	37 (7.27%)
… informal situation with an unknown man	147 (28.88%)	113 (22.20%)	165 (32.42%)	84 (16.50%)
… informal situation with an unknown woman	173 (33.99%)	100 (19.65%)	185 (36.35%)	51 (10.02%)

## Results

First, we conducted a 4 (actual facial hair: clean-shaven vs. light-stubble vs. heavy-stubble vs. full beard) × 3 (preferences related to facial hair: in oneself vs. in other men vs. preferred by women) mixed-model ANOVA × 2 (sample: convenience vs. internet panel). We observed an interaction of preferred facial hair and one’s own facial hair (*F*[6, 1002] = 12.99, *p* < 0.001, η_p_^2^ = 0.07; see Table 2). The more facial hair the participants had, the more facial hair they wanted for themselves. Participants with heavy stubble and full beards wanted more facial hair for themselves than for other men but clean-shaven participants wanted slightly less facial hair for themselves than for other men. Participants with heavy stubble and full beards wanted more facial hair for themselves than it was in their opinion attractive for women, but clean-shaven participants wanted less facial hair for themselves than it was in their opinion attractive for women. There were also main effects of actual facial hair (*F*[3, 501] = 38.23, *p* < 0.001, η_p_^2^ = 0.19) and preferences related to facial hair (*F*[2, 1002] = 14.88, *p* < 0.001, η_p_^2^ = 0.03). The more facial hair the participants had, the more facial hair they generally preferred. Participants preferred more facial hair for themselves (*M* = 2.81, *SE* = 0.05) than for other men (*M* = 2.48, *SE* = 0.05, *p* < 0.001), and it was, in their opinion, attractive for women (*M* = 2.69, *SE* = 0.06, *p* = 0.049); they also preferred less facial hair for other men than it was, in their opinion, attractive for women (*p* = 0.001)*.* There was also an interaction of actual facial hair and sample (*F*[3, 501] = 5.11, *p* = 0.002, η_p_^2^ = 0.03) and a main effect of sample (*F*[1, 501] = 9.90, *p* < 0.002, η_p_^2^ = 0.02). Cleanly shaven and heavily stubbled participants from the convenience sample preferred more facial hair than clean-shaven and heavy stubble participants from the internet panel sample (*p*s ≤ 0.040). Participants from the convenience sample generally preferred more facial hair than participants from the internet panel sample (*p* < 0.002).

**Table 2:** Marginal means [SEs] of effects related to facial hair preferences in the context of own facial hair

Actual facial hair	Preferences related to facial hair		
	For oneself	For other men	For women
Clean-shaven	1.96_ac_ [0.08]	2.16_a_ [0.09]	2.34_c_ [0.10]
Light stubble	2.24_a_ [0.11]	2.18_b_ [0.12]	2.56_ab_ [0.13]
Heavy stubble	3.18_ cd_ [0.08]	2.71_c_ [0.09]	2.78_d_ [0.10]
Full beard	3.87_ cd_ [0.12]	2.87_c_ [0.13]	3.08_d_ [0.14]

In rows means with a differ at *p* < .05, means with b differ at *p* < .01, means with c and d differ at *p* < .001

**Fig. 2:**
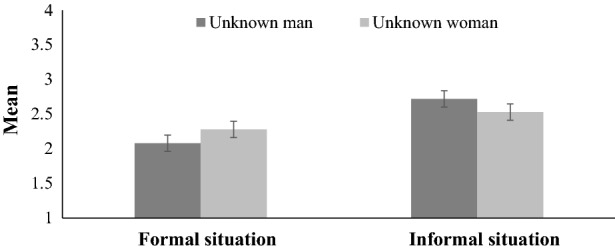
Perceived appropriate levels of facial hair in formal and informal situations with unknown persons. *Note*. Error bars are 95% confidence intervals

Second, we conducted a 2 (type of situation: formal vs. informal) × 2 (sex of the unknown person: man vs. woman) × 2 (sample: convenience vs. internet panel) mixed-model ANOVA with owned facial hair as a between-subjects factor. Situation and sex of an unknown person (*F*[1, 501] = 34.20, *p* < 0.001, η_p_^2^ = 0.06; see Fig. 2) and situation and facial hair (*F*[3, 501] = 3.87, *p* < 0.018, η_p_^2^ = 0.02) both interacted. The least appropriate was wearing facial hair in formal situations with unknown men, followed by formal situations with unknown women, informal situations with unknown women, and informal situations with unknown men. The more facial hair the participants had, the more they declared as appropriate in formal and informal situations; however, the declarations of cleanly shaven and lightly stubbled participants were similar. We also found main effects for actual facial hair (*F*[3, 501] = 33.57, *p* < 0.001, η_p_^2^ = 0.17) and the type of situation (*F*[1, 501] = 85.09, *p* < 0.001, η_p_^2^ = 0.15). Cleanly shaven and lightly stubbled participants perceived less facial hair as appropriate than those with heavy stubble and full beards (*p* < 0.001) and participants with heavy stubble perceived as appropriate less facial hair than those with full beards (*p* = 0.001). Less facial hair was perceived as appropriate in formal situations (*M* = 2.18, *SE* = 0.05) than informal situations (*M* = 2.62, *SE* = 0.05, *p* < 0.001). Situation and sample (*F*[1, 501] = 4.51, *p* < 0.034, η_p_^2^ = 0.01) and actual facial hair and sample (*F*[3, 501] = 4.42, *p* = 0.004, η_p_^2^ = 0.03) both interacted. Participants from the convenience sample perceived more facial hair than participants from the internet panel sample as appropriate in informal situations (*p* = 0.014). Cleanly shaven participants from the convenience sample perceived more facial hair than cleanly shaven participants from the internet panel sample as appropriate (*p* < 0.001).

## Discussion

Recent studies indicate that beards change men’s social perceptions regarding the aspects that are important in intrasexual rivalry among men (e.g., Dixson et al., 2021). Beards appear to (1) make men look more dominant and aggressive (Albert et al., 2020), (2) exaggerate facial expressions of anger (Dixson et al., 2021), and (3) compensate for a diminutive jaw size (Sherlock et al., 2017). Despite several studies about the impression management functions of facial hair, there is little research on men’s decisions, opinions, and preferences related to facial hair. Here, we attempted to understand whether men’s preferences regarding their facial hair are congruent with the impression management functions facial hair has demonstrated. We also investigated whether men’s judgments of appropriateness of facial hair are sensitive to social contexts.

We revealed two clear preferences toward facial hair. First, men reported higher preferences for their own facial hair compared to preferences for facial hair in other men. These results support an effect described in the previous studies (Jach & Moroń, 2020) which refers to preferences for less facial hair in other men compared to preferences for own facial hair. However, this difference appeared mainly among men with heavy stubble or full beards. In contrast, men with cleanly shaven faces wanted less facial hair for themselves than for other men. It appeared that only those men who have distinct facial hair manifest a preference for less facial hair in other men. This result could be interpreted in the context of the greater value attributed to features that occur rarely. Men with facial hair want their high-value display to be less frequent to enhance its impression management role as a unique attribute (Janif et al., 2014). Thus, men with distinct facial hair may want other men to have less facial hair which helps their own display of facial hair to appear as unique among men in each environment. On the other hand, men with light stubble would like the other men to have less facial hair compared to the facial hair they perceived as attractive for women. Thus, men without distinct facial hair might want other men to not have pronounced facial hair to attract women. This facial hair equalization could strengthen other strategic displays of their resources or appearance features and limit other men compensating for their mate value with facial hair (Mogilski & Welling, 2018). Therefore, men’s strategic display of resources or appearance could be accompanied by “background-setting preferences” which can help in creating a beneficial condition for a chosen display. These preferences may correspond to a role of context of other people for perceived attractiveness of a given person which is referred to such contrast effects as the cheerleader effect (McDowell & Starratt, 2019; Walker & Vul, 2014) or the friend effect (Ying et al., 2019). An individual’s attractiveness is perceived differently when the individual is seen alone than when is observed in a group (Lei et al., 2020). The perceived average facial attractiveness of a group influences the judgment of the target person implicitly thus people surrounded by unattractive friends may be perceived as more attractive (Ying et al., 2019). Therefore, men may be motivated to create a reference group of men who possess weaker cues of social dominance. For example, bearded men could prefer the company of less bearded men to enhance the distinctiveness of their facial hair as an attribute enhancing their perceived dominance and social position. The pattern of findings about men’s preferences toward beards could be also interpreted as an effect of a general desire to appear unique in an environment. Thus, future studies should investigate whether men’s preferences toward beards in the context of other men’s beardedness are unique or whether men have similar preferences toward skin color or jaw size.

Men’s preferences toward their own facial hair seem independent from their opinions about women’s preferences. These results are in line with an interpretation of facial hair as an attribute used in intrasexual rather than intersexual relationships (Dixson et al., 2017). Men prefer less or more facial hair compared to women’s perceived preferences depending on their actual facial hair. Cleanly shaven and lightly stubbled men revealed a preference for less facial hair, while men with heavy stubble and full beards preferred more facial hair in comparison to their estimated women’s preferences. However, lightly stubbled men also preferred less facial hair in other men compared to their estimated women’s preferences. Men who cannot display attractive facial hair may want to limit other men’s impression management through facial hair or limit the compensatory functions of facial hair relative to other qualities that influence judgments of mate value (Mogilski & Welling, 2018).

We also detected a pattern of men’s judgments of appropriateness of facial hair regarding the social context of its display. Facial hair was perceived as more appropriate for informal situations, especially with unknown men. Informal situations are characterized by fewer social norms about what is the appropriate way to look (de Souza et al., 2003) and the social attributions of certain appearance-related features (e.g., attributions linking beardedness with political beliefs; Herrick et al., 2015) than formal situations. Thus, informal, rather than formal, situations can be ecologically more equivalent to the naturally occurring situations that may have evolved intrasexual rivalry. In an informal interaction with another man, men indicated heavy-stubble or full beard as an appropriate image. Men infer from the beard of other men their dominance and aggressive intentions (Albert et al., 2020; Sherlock et al., 2017). Thus, displaying facial hair could instantly affect the position of a man in an interaction with another man; however, social customs demand restrained competitive intentions and arranging interpersonal interactions based on other criteria (e.g., professional). On the other hand, less favorable judgments of appropriateness of facial hair in formal situations can be a result of the ambiguous perception of men wearing beards in such situations, which are both beneficial (e.g., enthusiastic, sincere, generous; Kenny & Fletcher, 1973; Pellegrini, 1973) and unfavorable (e.g., giving less assurance, unconventional; Hellström & Tekle, 1994; Magnini et al., 2013).

### Limitations and Conclusions

In our study, we used an appropriate sample size and realistic pictorial stimuli to assess men’s preferences for facial hair and investigate the role of multiple contexts in judgments of appropriateness of facial hair. However, the current study is not without limitations. First, the pictorial scale of facial hair preferences includes only four categories of beardedness, which may be an oversimplification of the many ways men might adopt facial hair (Gray et al., 2020). Future studies may investigate preferences for specific modes of expression of facial hair. Moreover, studies are needed on the grooming and trimming of facial hair as particular measures of appearance enhancement. In hiring decisions, trimmed and groomed beards were assessed as signs of competence (Reed & Blunk, 1990), but other studies that did not explicitly control for the specific image of a beard indicated an unfavorable effect of beards on hiring decisions (de Souza et al., 2003). Second, it would also be worth controlling such a variable as the level of cranial hair of the presented models because the presence or absence of hair on male faces affects the perception of their age and such social characteristics as aggressiveness, appeasement, social maturity, and attractiveness (Muscarella & Cunningham, 1996). In our study, we asked subjects for their opinions on facial hair, not entire faces; however, their declarations may have been affected by the general appearance of the photos. Future studies should also use numerous models to control for variance connected with cranial shape and other attributions of face shape which may confound the judgments about facial hair (e.g., attribution of higher attractiveness to averaged faces; DeBruine et al., 2007). Third, future studies can also control for the frequency of facial hair in the general population to better examine the frequency-dependent effects (e.g., negative frequency-dependent preferences; Janif et al., 2014). Fourth, research should also monitor what participants understand as formal or informal situations and what types of formal and informal situations occur in their lives. Fifth, we assessed how men’s current beardedness related to their preferences and judgments of beards, but this ignores motivations for having the beard in the first place or how those motivations might mediate men’s opinions about facial hair. For instance, dispositional tendencies toward intrasexual competition (Arnocky & Carré, 2016; Buunk & Fisher, 2009), personality traits like psychopathy, Machiavellianism, and narcissism (Bird et al., 2016; Jonason et al., 2020), and motivational biases of wanting power or affiliation (Carpinella & Johnson, 2013; Mannes, 2012; Sherlock et al., 2017) may also play important roles in the psychology surrounding beards. Sixth, our results focused on Polish men only but there may be a reason to examine intrasexual competition, and thus beardedness, across cultural contexts (Buunk, 2022). Moreover, human populations vary in facial hair growth (e.g., facial hair is a more characteristic feature for European and Central Asia populations than African populations and East-Asian populations; Pazhoohi & Kingstone, 2020), so our results may pertain more to some populations than other populations. While we included two types of samples, a more robust, theory-driven test of the functions of beards as a function of country-level factors like the operational sex ratio, pathogens, and harshness may prove fruitful to understand hypotheses regarding beardedness whereas gender equality indexes may bear on feminist approaches to beardedness in men (e.g., Dixson & Lee, 2020; Marcinkowska et al., 2019). Such a methodology would better check whether the obtained results are related to the universal impression management potential of beards or only to cultural display rules in a single (e.g., Polish) culture. Seventh, our convenience sample had more facial hair and more favorable opinions about facial hair than the internet panel sample. However, these differences may reflect the impression management functions of facial hair because participating in a survey on facial hair without remuneration may be more attractive to men with facial hair and with more positive attitudes towards facial hair. Lastly, the present study investigated men’s preferences and judgments according to beards based on the results showing that facial hair affects the social perception of men’s characteristics. Future studies should investigate whether wearing facial hair is indeed associated with higher aggression, dominance, social status, father abilities, and other traits. Moreover, future studies should also investigate whether beardedness is an honest signal in terms of signaling theory of sexually dimorphic traits (Zahavi, 1975). Thus, the costs of beardedness and its associations with health status, physical strength, and body shape should be examined in order to determine whether impression management functions of facial hair also reflect its signaling role (for a similar approach regarding voice pitch see Aung & Puts, 2020).

Despite these limitations, we revealed that men prefer other men to have less facial hair, especially men with heavy-stubble and full beards wanted other men to display less facial hair. In addition, facial hair was perceived as more appropriate in informal situations with unknown men. Our results suggest that men may be aware of the impression management functions of their facial hair. These results are also in line with the suggestion that facial hair among men is a display strategically deployed in intrasexual rivalry rather than in intersexual relationships.

## Supplementary Information

Below is the link to the electronic supplementary material.

Supplementary file1 (XLSX 38 KB)

## Data Availability

Data have been included as Supplementary material.
